# Thirty years of experience with anti-neutrophil cytoplasmic antibody glomerulonephritis in Charles Nicolle Hospital-Tunisia: a retrospective cohort study

**DOI:** 10.11604/pamj.2022.42.84.27914

**Published:** 2022-06-01

**Authors:** Meriam Hajji, Samia Barbouch, Rim Goucha, Fethi Ben Hamida, Imen Gorsane, Ezzeddine Abderrahim

**Affiliations:** 1 Department of Medicine A, Charles Nicolle Hospital, Tunis, Tunisia,; 2 Faculty of Medicine of Tunis, University of Tunis El Manar, Tunis, Tunisia,; 3 Laboratory of renal pathology LR00SP01, Charles Nicolle Hospital, Bab Saadoun, Tunis, Tunisia

**Keywords:** Crescentic glomerulonephritis, ANCA, prognosis

## Abstract

**Introduction:**

antineutrophil cytoplasmic antibodies (ANCA) associated Glomerulonephritis (GN) is rare but a life-threatening disease especially, particularly in patients with advanced renal failure at presentation. This study aims to evaluate the epidemiological, clinical and histopathological features of renal involvement and investigate factors associated with ESRD.

**Methods:**

patients with renal biopsy-proven ANCA associated glomerulonephritis were included retrospectively over a thirty years period. The renal survival, defined as time to reach ESRD, was evaluated based on clinical parameters, histopathological classification as well as the renal risk score.

**Results:**

a total of 65 patients with crescentic GN were included in the study. The mean age was 47.9 years ± 22.4 years (range: 18-78) with an M/F sex ratio at 1.13. Hematuria, proteinuria and oliguria were found in respectively 100%, 81.5% and 56.2% of cases. Sixty patients (92.3%) had renal failure at presentation, and 30 patients (46%) required initial hemodialysis (HD) therapy. The pattern of glomerular injury was categorized as mixed in 43.7% of cases, sclerotic in 34.3%, crescentic in 16.6%, and focal class in 6%. Regarding renal risk score, patients were classified in the category low risk, intermediate risk and high risk with respectively 16.9%, 44.6% and 38.4%. All patients received corticosteroids and immunosuppressive treatment. Complete, partial remission and relapses were noted in respectively 15.3%, 18% and 72% of cases. Factors associated with ESRD were serum creatinine level >500 μmol/l (P=0,0016), CRP level >60 mg/l (P = 0,0013), interstitial fibrosis (P=0,0009) and glomerulosclerosis> 10% of total glomeruli (P=0,001). The survival rate was 89%, 60.9% and 32.8% at respectively 1, 5 and 10 years. Death occurred in 10 cases (15%) caused mostly by infections (40%). Initial serum creatinine level>140 μmol/l (P=0,02), alveolar hemorrhage (P=0.001) and infections (P=0,0001) were associated with mortality.

**Conclusion:**

in our cohort of ANCA GN, confirms the data showing improved patient survival but constantly high relapse risk. In addition, we observed that ANCA GN classification was predictive, as the risk of progressing to ESRD increased with the ascending category of focal, crescentic, mixed and sclerotic GN.

## Introduction

Pauci-immune glomerulonephritis (GN) is a rare group that occurs as a renal-limited disease causing aggressive GN or as a component of a systemic necrotizing small-vessel vasculitis. It is characterized by paucity of staining for immunoglobulins (Ig), by immunofluorescence (IF) along with fibrinoid necrosis and crescent formation by light microscopy [1]. Renal histological study is crucial for not only the diagnostic value, but also for therapeutic and prognostic impact. In 2010, Berden et al. proposed a histological classification that ranks ANCA associated GN into four classes: focal, crescentic, mixed, and sclerotic, and it was initially shown that these categories were correlated to renal outcome [2,3]. Therefore, we will focus on the prevalence and particularities of pauci-immune GN in our department and therapeutic options and prognosis for these diseases. In 2018, Brix et al. proposed a clinically applicable renal risk score that allowed early risk prediction of end-stage renal disease (ESRD) [4]. In the present study, we analyzed clinical and histological findings of 65 patients with ANCA-associated GN and assessed the prognostic impact of histological classification and renal risk score on renal and overall survival in our population.

## Methods

**Study design:** we conducted an analysis of retrospectively collected data of 65 adult patient´s biopsy proven pauci-immune GN, over a thirty years period. These patients were referred to our nephrology department either for exploration of an acute renal failure or a rapidly progressive GN or a pulmonary renal syndrome. The diagnosis of ANCA vasculitis was retained, based on the Chapel Hill consensus [5].

**Setting:** hospital charts were manually reviewed to identify patients with a clinical diagnosis of renal vasculitis. Patients were selected for inclusion on the study only if they had histologically confirmed renal disease and a full documented follow-up in our department, from clinical diagnosis to evolution after initiation of treatment. Were excluded patients with secondary vasculitis forms. The renal survival, defined as time to develop ESRD requiring dialysis initiation, was evaluated based on clinical parameters, the histopathological classification [2], and renal risk score [4].

**Study population:** we enrolled 65 patients with ANCA associated GN, including 27 with microscopic polyangiitis (MPA) and 37 with granulomatosis and polyangiitis (GPA)while the last patient was a young man aged 50 years old and was diagnosed with eosinophilic granulomatosis with polyangiitis (EGPA).

**Data collection:** demographic, clinical presentation, histopathological, treatment specifications and follow-up parameters were collected. All patients had been tested for the presence of ANCA by indirect immunofluorescence.

**Study procedures:** the ANCA assay became available for clinical use in our hospital since 1985. For the purpose of classification, in the late 1990s, ANCA tests were performed using only indirect IF microscopy and patients were classified as either C-ANCA (cytoplasmic) positive or P-ANCA (perinuclear) positive. Since 2000, the detection of autoantibodies using enzyme-linked immunosorbent assay (ELISA) reacting with proteinase 3 (PR3) or myeloperoxidase (MPO) was available in our hospital. Renal specimens were evaluated using light microscopy and direct IF. For light microscopy, paraffin sections were stained with silver, periodic acid-Schiff, hematoxylin eosin and, trichrome. Then, they were forwarded to two expert nephropathologists in our department. The IF studies were performed using antibodies against IgA, IgG, IgM, C3, C1q, fibrin, albumin, and k and l light chain. A minimum of eight glomeruli was considered adequate for a renal biopsy to be included. Each glomerulus was scored separately for the presence of fibrinoid necrosis, crescents (cellular/fibrous/fibro cellular), glomerulosclerosis (segmental/global), granulomatous reactions, and endocapillary and mesangial cellular proliferation. The presence of glomerular lesions was calculated as the percentage of the total number of glomeruli in a biopsy. Glomerular lesions were assigned to four categories according to the definition of the 2010 histological classification [3]. Tubulointerstitial lesions such as interstitial fibrosis and tubular atrophy and interstitial inflammation were graded semi- quantitatively (1 for < 25%, 2 for > 25% and < 50%, and 3 for > 50%). Vascular lesions were scored as present or absent.

### Definitions

Hematuria: is the presence of 5 or more red blood cells per high-power field in 3 of 3 consecutive centrifuged specimens obtained at least 1 week apart. Oliguria is defined as a urine output that is less than 400 ml daily. Anuria is defined as a urine output that is less than 100 mL daily. Glomerular filtration rate (GFR) was estimated according to the Modification of Diet in Renal Disease formula [6]. Disease activity was scored using the Birmingham Vasculitis Activity Score (BVAS) [7].

ESRD: creatinine clearance < 10 mL/min/1.73 m^2^ and/or the use of chronic renal replacement therapy. The renal risk score (RRS) included three parameters [4]: Percentage of normal glomeruli (N): N0 >25%, N1 10%-25%, N2 < 10%. Percentage of interstitial fibrosis and tubular atrophy (T): T0 = 25%, T1 > 25%. GFR at the time of diagnosis (G): G0 =15 mL/min/1.73 m^2^, G1 < 15 mL/min/1.73 m^2^. N1: 4 points, N2: 6 points, T1: 2 points, G1: 3 points. The resulting score was used to classify predicted ESRD risk at 36 months as low (0 point: 0%), intermediate (2-7 points: 26 %), or high (8-11 points: 68%-78%) Complete remission: normalization of renal function and disappearance of urinary sediment abnormalities associated with the resolution of extra-renal manifestations.

Partial remission: an improvement without normalization of the renal function and/or stabilization of the renal function, as well as the resolution of extra-renal manifestations. Relapse: A reactivation of the disease following a remission. It can manifest as a rapid deterioration of renal function associated with abnormal urinary sedimentation and/or worsening or onset of new extra-renal signs.

**Statistical analysis:** to investigate predictive factors for renal relapse and prognostic factors of renal and overall survival, we used SPSS 20.0 for Windows (SPSS Inc, Chicago, IL) software. We calculated simple frequencies and relative frequencies (percentages) for qualitative variables. We calculated averages, medians and standard deviations and determined the extreme values for the quantitative variables. The results were presented in the form of summary tables. Comparisons of 2 averages on independent series were made using Student´s test t for independent series. Comparisons of 2 averages on matched series, and in the case of numbers < 30, were made by the nonparametric Wilcoxon test. The comparisons of percentages on independent series were made by the Pearson Chi-square test, and in the case of non-validity of this test, and by a comparison of two percentages, by the test of Fisher. The links between two quantitative variables were studied by the Pearson correlation coefficient and in the case of non-validity by the correlation coefficient of the ranks of Spearman. Univariate analysis was performed by calculating the Odds ratio, using the log rank test. Survival data were studied by establishing a survival curve according to the Kaplan Meier method. A multivariate analysis was performed using Cox regression. In all statistical tests, a P value of less than 0.05 was considered statistically significant.

**Ethical considerations:** the anonymity of study subjects was respected during the data collection.

## Results

Our study population was mean aged 47.9 ± 22.4 years (range: 18-78). The female-to-male ratio was 1.13. Fifty-seven of our patients (87%) were from the north and the center of the country. Forty-five patients were hospitalised through the emergency department (69.2%), while the rest were transferred through other hospital departments as pneumology, internal medicine and ENT with respectively (30%, 50% and 20%). General characteristics of patients are presented in Table 1. The mean level of BVAS at diagnosis was 17.4 ± 6.5 (11-39). The prevalence of extra-renal organ involvement was different between both groups (Table2). The presence of Ear, nose and throat (ENT), central nervous system, cardiac, digestive and ophthalmic involvement among patients with GPA was significantly higher while pulmonary and peripheral nerve involvement was significantly higher in MPA patients (Table 2). All of our patients were ANCA positive in indirect IF assay, whereas ELISA was performed in only 42 cases (64.6%). Twenty-eight patients (75.6%) with GPA were tested positive for PR3/c- ANCA, and twenty-five patients (92.5%) with MPA were tested also positive for MPO/p-ANCA. For the one patient diagnosed with EGPA, he presented pANCA positivity. Among GPA patients, two had concomitantly p-ANCA and c-ANCA positivity. Among c-ANCA patients, five also showed anti-glomerular basement membrane (anti-GBM) antibodies. In our cohort, renal manifestations were dominated by microscopic hematuria found in all cases, oligo-anuria was noted in 5 patients (7.7%) and 20 patients (30.7%) had hypertension. Fifty-two patients had proteinuria (80%), nine of which had nephrotic syndrome (17.3%). Mean baseline serum creatinine was 664μmol/l (90-1040) with a mean Cr clearance at 12.7ml/min/1.73m^2^ (4-92). Sixty patients (92.3%) had renal failure at presentation.

**Table 1:** general characteristics of our study population

	N (%)	GPA (n)	MPA (n)
**Age range**			
<35 years old	10(15)	6	4
]35-65[ years old*	49(75)	27	21
> 65 years old	6(9)	4	2
**Gender**			
Men*	35	18	16
Women	30	19	11
Smoking	29 (44.6%)	18	10
**Medical history**			
Diabetes	11 (17)	8	3
Hypertension	20 (31.2)	11	9
Cardiovascular disease	4 (6)	2	2
**Mode of onset of the disease**			
Acute kidney failure	4(6.1)	3	1
Pulmonary renal syndrome	6(9.2)	2	4
Rapidly progressive GN	55(84.6)	32	22
Diagnostic delay (months)	10.3±5	-	

N, n= number of patients, *one patient was diagnosed with EGPA, GN: glomerulonephritis

**Table 2:** extra renal manifestations seen in our patients

	N(%)	GPA	MPA	P
**General signs:**				0.08
Long-term fever	6(9.3)	2(5.4)	4(14.8)	
Anorexia and slimming	42(64.7)	26 (70)	16(59)	
**Pulmonary involvement:**				
Hemoptysis	18(28.6)	5(13.5)	13(48)	**0.009**
Dyspnea	15(23)	6(16.2)	9(33.3)	0.06
Cough	7(10.7)	4(10.8)	3(11.1)	0.1
**ENT involvement:**				**0.005**
Epistaxis	5(7.7)	3(8.1)	2(7.4)	
Otitis	2(3)	2(5.4)	-	
Sinusitis	4(6.1)	4(10.8)	-	
Crusty rhinitis	1(1.5)	1(2.7)	-	
**Neurologic involvement:**				
Headaches	9(13.8)	5(13.5)	4(14.8)	-
Neurologic allocalization signs	5(7.7)	5(13.5)	-	**0.02**
Lowerlimb paresthesia	5(7.7)	1(27)	4(14.8)	**0.01**
Psychic disorders	2(3)	2(5.4)	-	-
**Articular involvement**				0.07
Arthralgia	14(21.5)	4(10.8)	10(37)	
Arthritis	-	-	-	
**Cutanous involvement**				0.08
Vascular purpura	9(13.8)	7(18.9)	2(7.4)	
Skin-in-skin nodos	1(1.5)	1(2.7)	-	
**Cardiac involvement**	20(30.7)	8(21.6)	3(11.1)	**0.001**
**Ocular involvement**	9(13.8)	7(18.9)	2(7.4)	**0.04**
**Gastro-intestinal involvement**	4(6.1)	4(10.8)	-	**0.04**

GPA: Granulomatosis with polyangiitis; MPA: Microscopic polyangiitis, N: Number

**Figure 1:**
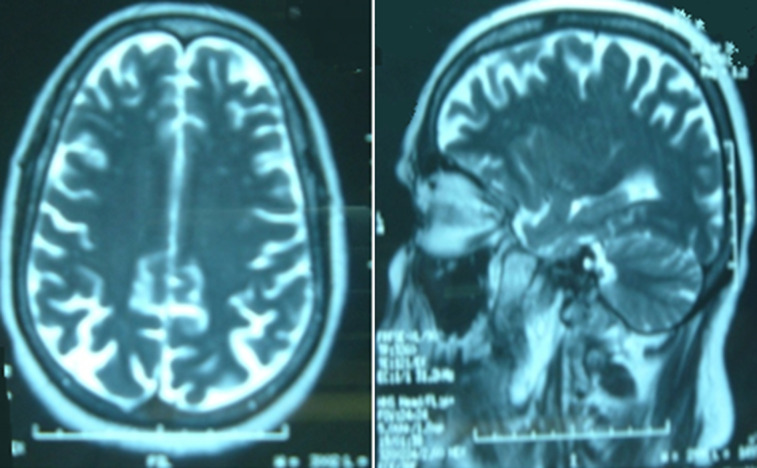
MRI findings of cerebral vasculitis including multiple supratentorial demyelination lesions in a patient diagnosed with granulomatosis with polyangiitis in our study population

Regarding histological manifestations, patients had a median of 24 (10-53) glomeruli per biopsy specimen. A detailed histologic analysis of biopsies was performed and presented in Table 3. We noted a predominance of the mixed class (43.7%). The IF staining revealed a pauci immune GN in all cases. Table 4 illustrates our study population characteristics on the basis of histological class. The treatment, instituted in our patients, was corticosteroids combined with cyclophosphamide (CYC). For induction therapy, all patients received intravenous (IV) methylprednisolone (MP) pulse therapy at a dose of 15 mg/kg daily for three days followed by oral corticosteroids (Cst) at a dose of 1 mg/kg/day. IV CYC was administered at the dose of 500 mg/m^2^ bimonthly in respectively 9/37 patients with GPA and 5/27 patients with MPA and monthly in 28/37 patients with GPA and 22/27 patients with MPA, for 6 months duration. Plasma exchanges (PE) were performed in five cases (7.6%) with alveolar hemorrhage (AH) in 4 cases and with documented cerebral vasculitis in 1 case. Four patients (6%) were additionally treated with IVIg for recurrent episodes of AH despite immunosuppressive treatment and PE. One patient with GPA was treated by IV Ig for a documented cerebral vasculitis (Figure 1).

**Table 3:** renal histopathological findings in our study population

Histologic signs	N (%)
**Percentage of normal Glomeruli**	
N0	20
N1	28
N2	16
**Glomerular lesions**	
Cellular crescents	25(39)
Cellulofibrous crescents	18(28)
Fibrous crescents	21(32)
Glomerular necrosis	24(37.5)
Capillary rupture	10(15.6)
Cb rupture	14(21.8)
Periglomerular granuloma	10(15.6)
Fibrinoidnecrosis	24(37.5)
**Tubulo interstitial lesions**	
Inflammatory interstitium (>50%)	12(18.7)
Acute tubular necrosis	10(15.6)
Hematic cylinders	54(84.3)
Interstitial fibrosis (>25%)	11(17.1)
**Vascular lesions**	
Thrombotic microangiopathy	3(4.6)
Vasculitis	7(11)
Fibrous endarteritis	10(15.6)

GPA: granulomatosis and polyangiitis, MPA: microscopic polyangiitis, ENT: Ear Nose and Throat, N0: >25%, N1: ]10%-25%[, N2: <10%

**Table 4:** clinical characteristics according to Berden histological classes

	Berden Histological classes				P
	focal	Crescentic	mixed	sclerotic	
Number (%)	4(6)	10(16.6)	28(43.7)	22(34.3)	
Mean Age	32±8	45±6	52±11	58±9	-
Sexe ratio M/F	2/2	6/4	13/15	13/9	-
BVAS	11	18	26.5	22.5	**0.01**
GPA/MPA	3/1	5/5	17/11	12/10	-
Mean GFR (ml/min)	37.2±6	23.4±5	13.5±4	4.7±2	**0.01**
Hemodialysis at diagnosis (n)	0	4	12	14	0.22
Median Proteinuria (g/24h)	0.57	1.1	1.7	1.64	0.12
Median Hemoglobine (g/dl)	10.4	9.6	8.2	7.7	0.1
Median PaO2	82	75.1	72.4	76.6	**0.08**
Mean Gold score (AH)	130±20	250±19.5	270±11.7	290±8.2	0.3
Median CRP (mg/l)	55.5	60	38.6	32.3	**0.002**
ESRD at the end of the study (n)	0	2	9	17	-

BVAS: Birmingham Vasculitis Activity Score, GPA: granulomatosis polyangeitis, MPA: microscopic polyangeitis, GFR: glomerular filtration rate, PaO2: partial pressure of oxygen, AH: Alveolar haemorrhage, ESRD: end stage renal disease CRP: C-reactive protein

Initial hemodialysis (HD) was necessary in 30 patients (46%). The maintenance treatment was carried out with azathioprine (AZA) in 14 cases (21.8%) and with my cophenolatemofetil (MMF) in 5 cases (7.8%). Forty-six patients did not benefit of a maintenance treatment because of ESRD onset in 27 patients (41%), the loss of follow-up in 9 cases and the occurrence of death in 10 cases. The median follow-up period was 84.8 ± 10 months (range: 13-156). Complete remission was noted in 18 cases (28%) and partial remission in 12 cases (18%). Relapses were noted in 32 cases (49%) after a mean period of 9.4 months (range: 7-23). The follow-up period was marked by Thrombo-embolic accidents in 7 patients (10.7%). To note, a thrombophlebitis of the transverse sinus occurred in a patient with GPA. Fifteen patients (23%) developed infectious complications which consisted in pneumonia in 11 patients (17%), endocarditis in 2 patients and purulent sinusitis in 2 cases.

At the end of our study, Twenty-eight patients (43%) developed ESRD. Regarding renal risk score, we had 11 patients (16.9%) with low risk, 29 with intermediate risk (44.6%), and 25 (38.4%) with high risk. ESRD occurred in 11 patients (37.9%) of intermediate-risk group and 17 patients (68%) of high-risk group. In our study, we found, in univariate analysis, that the following factors were significantly associated with the occurrence of ESRD: initial serum creatinine (>500 μmol/l) (P= 0.001) and high-rate CRP level (>60 mg/l) (P=0.009). In the multivariate analysis, serum creatinine at diagnosis, CRP (>60 mg/l) and sclerotic class remained as significant predictors of ESRD (P= 0.016; P= 0.019; P= 0.001 respectively). Predictive factors for relapse have been identified as ANCA positivity at 6 months (P=0.02) and infectious complications (P=0.03). A statistically significant association with discontinuation of immunosuppressive therapy (P=0,003) has also been demonstrated. Finally, two mortality predictive factors were determined: alveolar hemorrhage (P=0.001) and infectious complications (P=0.0001).

## Discussion

ANCA associated vasculitis are considered as a rare disease with an incidence of about 20 per million population per year in Europe and North America [8]. There is a notable geographic distribution, with GPA being more common in Northern Europe, whereas MPA is more common in Southern Europe and Asia [9]. There have increased in prevalence since the 1980s [10-12]. African epidemiological data of ANCA vasculitis remain limited and are likely to be related to the predominance of infectious diseases and socio-economic problems [13]. It is unclear whether this represents genetic differences or other environmental factors such as vitamin D levels and sun exposure. There are only very few clinical studies on ANCA vasculitis from Africa, and still less on renal involvement. ANCA vasculitis have a predilection for the kidney, with > 75% of patients having a rapidly progressive GN. Between 2000 and 2020, in Tunisia, there have been small published series [14-16]. Renal involvement in ANCA vasculitis, is frequent, ranging from 80% to 97% according to various studies [17-20]. The typical renal presentation is that of a rapidly progressive GN with a decline in kidney function accompanied by low range proteinuria, microscopic hematuria, and hypertension over days to a few months [21,22].

**Figure 2:**
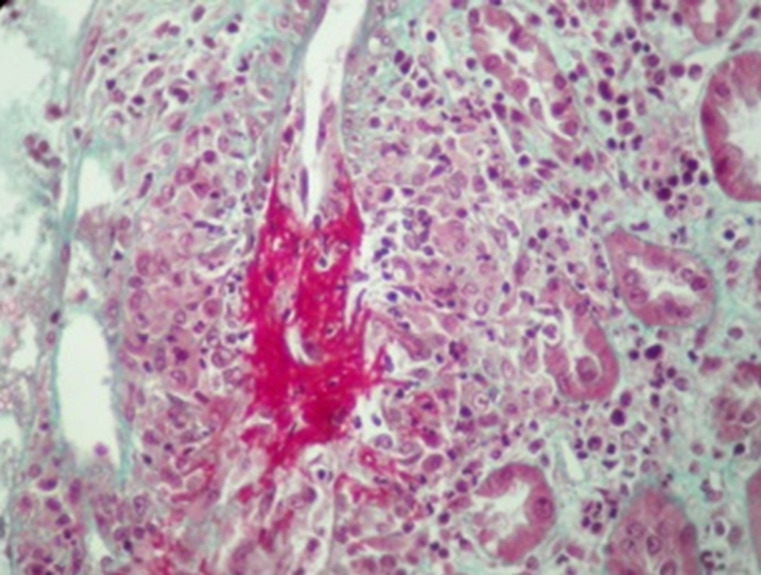
interlobular arterial vasculitis with fibrinoid necrosis (Trichrome de Masson x 400)

In our study, it is a 100% of renal involvement including patients with severe renal involvement, given the selection bias that we are a nephrology department. Histologically, pauci-immune necrotizing and crescentic GN is the typical pattern of glomerular injury in all forms of systemic ANCA associated vasculitis [8]. The areas of necrosis maybe small and segmental, or maybe more extensive with large circumferential crescents. Occasionally, these can rupture the Bowman capsule, provoking a brisk tubule interstitial inflammatory response [23,24]. Less commonly, patients may show extra glomerular renal vasculitis [8], which was found in 7 patients of the study population (Figure 2). For the IF study, although pauci-immune, small amounts of IgG or C3 may be seen and if present, have been associated with more severe disease [25]. We noted, in our patients, a predominance of the mixed class (43.7%). However, a caveat in Berden *et al*. classification is that in patients with GFR < 15 ml/min, the histological class does not predict renal outcome [26]. In this setting, normal glomeruli < 10% and higher overall chronicity score are risk factors for ESRD. In our study, only the sclerotic class was associated with progression to ESRD (P= 0.001). Indeed, 46% of our patients initially required HD. Our results are consistent with many studies that correlate histological classification to the renal prognosis [27-30].

Regarding extra-renal manifestations in literature, they are prominent and maybe present for several months before presentation. The lungs are more commonly involved [31,32] which agrees well without results, considering respiratory involvement was the most frequently reported manifestation in our study(61.5%). It is important to note that this last, is less common in GPA and lung involvement in MPA typically presents as AH and maybe associated with pulmonary fibrosis [22]. In our study, AH was noted in 5 versus 13 patients, GPA/MPA (p=0.009). Peripheral neuropathy, typically mononeuritis multiplex, occurs, but central nervous system involvement is rare [23]. It should be noted that five among our GPA patients had manifested neurological localization signs. Classifying patients on the basis of PR3-ANCA versus MPO-ANCA, correlates with a number of disease characteristics [27]. In our series, all of our patients were ANCA positive in indirect IF assay. ELISA was performed in 64.6% of cases because of the lack of the appropriate laboratory facilities in the 1980s. Among c-ANCA patients, five also showed anti-glomerular basement membrane (anti-GBM) antibodies. This was reported in literature, with approximately 5% of patients with ANCA vasculitis that also have anti-GBM antibodies, and approximately 35% of patients with anti-GBM disease have ANCA (usually MPO-ANCA) [33]. Among GPA patients, two had concomitantly p-ANCA and c-ANCA positivity, and it should be noted that those two patients had developed AH.

For decades, conventional treatment of ANCA associated vasculitis has been with high-dose CYC and Cst, which has induced remission in approximately 75% of patients at 3 months and up to 90% at 6 months, although relapses and adverse side effects were frequent [34]. All of our patients received immunosuppressive treatment, that combined Cst and CYC, which is in agreement with KDIGO guidelines since the presence of organ-threating manifestations [12]. PE were performed in five cases. The basis for considering PE in ANCA GN, is that the removal of ANCAs and other inflammatory mediators can promote earlier reversal of the immunologic response and minimize tissue damage, but it remains controversial [35,36]. Four patients (6%) were additionally treated with IV Ig. Through different studies, therapeutic effects of IVIG in patients with ANCA associated vasculitis have not been established so far. Some of them found that IV Ig was associated with rapid improvements in disease activity and the related biomarkers in patients with active vasculitis [37,38]. Recent Randomized controlled trials, have refined the therapy of ANCA associated vasculitis and transformed this group of diseases from a fatal disease to a chronic one with frequent relapses and associated in an iatrogenic morbidity [39-43]. However, in our study, most of our patients often have a delay in diagnosis and treatment, because of the delay in referral to a nephrology is firstly, then by the patients themselves not going to physicians at the onset of symptoms and in this case, they are mostly hospitalised through the emergency department (69.2%).

Regarding prognostic factors, kidney disease was reported as the most important predictor of mortality [44]. Those who present with GFR < 50 mL/min have a 50% risk for death or kidney failure at 5 years [45]. Our analysis results found that, creatinine at diagnosis, and sclerotic class are predictors of ESRD (P= 0.016; P= 0.001). Hence, Infectious complications were predictive factors for relapse (P=0.03). Some previous studies have also found that between Cst exposure and infection is well established [46,47]. Our series is limited by the fact that it is retrospective, but those data deserve to be considered, seeing the large number of patients collected and the long follow-up time of 30 years and also the diagnosis based on renal biopsy in all patients, in one of the largest nephrology departments in Africa. Our study certainly should be confirmed by a multicentre prospective study and the ongoing challenge, is to define the factors associated with a higher relapse risk and individualize the maintenance therapy accordingly.

## Conclusion

Our study indicates that ANCA associated GN is an important determinant not only of a patient’s renal prognosis but also his survival. Thus, renal involvement is one of the pillars to be considered in the therapeutic decision. Timely diagnosis and institution of appropriate immunosuppressive therapy is critically important for optimum renal outcome in patients with ANCA GN.
